# Necroptosis in head and neck squamous cell carcinoma: characterization of clinicopathological relevance and in vitro cell model

**DOI:** 10.1038/s41419-020-2538-5

**Published:** 2020-05-22

**Authors:** Jingyuan Li, Sihui Huang, Lijuan Zeng, Kan Li, Le Yang, Siyong Gao, Chenyu Guan, Sien Zhang, Xiaomei Lao, Guiqing Liao, Yujie Liang

**Affiliations:** Department of Oral and Maxillofacial Surgery, Guanghua School of Stomatology, Guangdong Provincial Key Laboratory of Stomatology, Sun Yat-sen University, Guangzhou, Guangdong People’s Republic of China

**Keywords:** Head and neck cancer, Necroptosis

## Abstract

Necroptosis is a recently discovered form of programmed cell death (PCD) having necrotic-like morphology. However, its presence and potential impact with respect to head and neck squamous cell carcinoma (HNSCC) are still unknown. The aim of this study was to reveal the necroptosis status and its clinicopathological relevance in HNSCC and to establish an in vitro model. We first analyzed the level of p-MLKL, MLKL, and tumor necrosis in HNSCC patient tissues as well as their correlation with clinicopathological features. Results showed that approximately half of the tumor necrosis can be attributed to necroptosis, and the extent of necroptosis is an independent prognostic marker for patient’s overall survival and progression-free survival. Then we established and thoroughly verified an in vitro model of necroptosis in two HNSCC cell lines using combined treatment of TNF-α, Smac mimetic and zVAD-fmk (TSZ). At last, we adopted this model and demonstrated that necroptosis can promote migration and invasion of HNSCC cells by releasing damage-associated molecular patterns. In conclusion, our study unveiled the necroptotic status in HNSCC for the first time and provided a novel in vitro model of necroptosis in two HNSCC cell lines. In addition, our results indicated that necroptosis may be a potential cancer promoter in HNSCC. This study may serve as the foundation for future researches of necroptosis in HNSCC.

## Introduction

Head and neck squamous cell carcinoma (HNSCC) is one of the most prevalent cancers worldwide. It was responsible for ~800,000 newly diagnosed cases and >400,000 cancer-associated deaths in the year 2018^1^. Even though there have been significant advances in the multimodal treatment of HNSCC, the long-term survival rates are still between 40 and 50%. Further improvements in therapeutic strategies are needed especially for the patients with advanced-stage cancers^2^. Resistance to chemotherapy is considered as one of the main reasons for the treatment failure in HNSCC. Previous studies have demonstrated that tumor cell apoptotic resistance is one of the principal mechanisms behind chemoresistance^3^. Traditionally, researchers sought to enhance the chemosensitivity through inhibition of antiapoptotic processes such as IAPs’ function and autophagy in tumor cells^4–9^. However, none of these methods have helped to overcome the apoptotic resistance and chemoresistance effectively.

Necroptosis is a recently discovered form of programmed cell death (PCD)^10^. In contrast to apoptosis here cells show necrotic features such as membrane permeabilization, cell swelling, and release of damage-associated molecular patterns (DAMPs)^11^. Classic necroptosis pathway is initiated by the activation of receptor interacting protein kinase-1 (RIP1), which binds and phosphorylate downstream RIP3 to generate a complex called necrosome. p-RIP3 subsequently phosphorylate MLKL, which then oligomerized and translocated to the plasma membrane. The pore-forming ability of phosphorylated MLKL then disrupts the integrity of plasma membrane and causes cell death accompanied by release of intracellular components such as DAMPs. These intracellular components often serve as danger signals released by dead cells and can induce strong inflammatory response^12^.

Under normal conditions, necroptosis is considered as a back-up for apoptosis as caspase 8 is a strong inhibitor of this process^11^. However, *CASP8* has been demonstrated by several researchers to be one of the most frequently mutated genes and an essential factor that can cause apoptosis resistance in HNSCC^13,14^. Therefore, targeting necroptosis may present a novel strategy that can bypass the apoptotic resistance and eliminate tumor cells in HNSCC^15^.

Necrosis is a prevalent pathological phenomenon in most of the solid tumors^16^ including HNSCC. The discovery of necroptosis raised a series of intriguing questions such as: is the necrosis in HNSCC can be fully or partially attributed to necroptosis? What is the role of necroptosis in HNSCC? Is it possible to manipulate the associated signaling cascade for improving HNSCC treatment? Unfortunately, no studies related to necroptosis in HNSCC are currently available also it is poorly understood in other cancers. Therefore, the main aim of this preliminary study is to reveal the necroptosis status and its clinicopathological relevance in HNSCC. We have also tried to establish and validate a cellular model of necroptosis in HNSCC.

## Results

### Necrotic foci observed in HNSCC tumor tissues are partially necroptosis

**Fig. 1:**
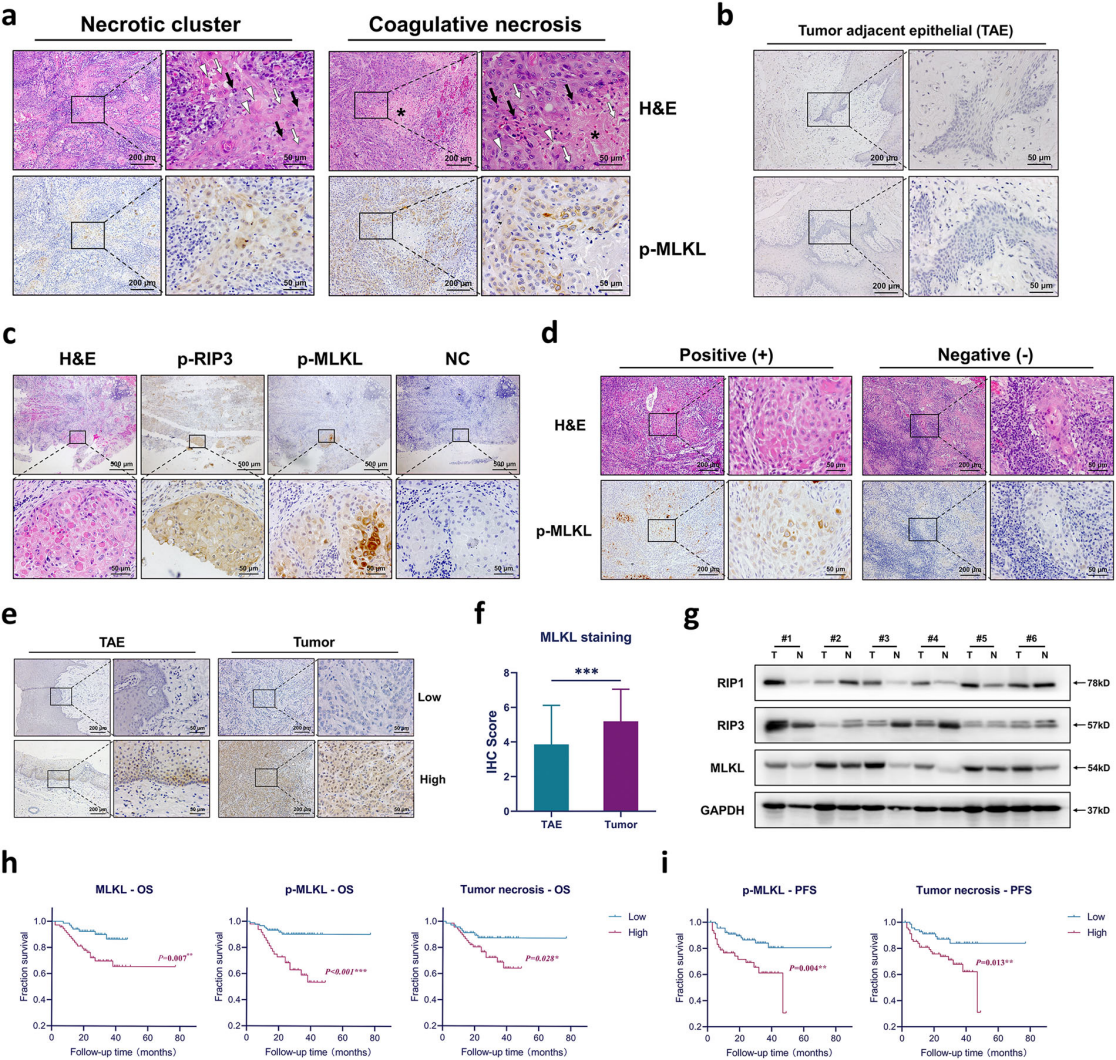
Necroptotic status in HNSCC patients and its clinicopathological relevance. **a** Staining pattern of p-MLKL in HNSCC tumor tissues and the corresponding H&E sections. The necrotic morphologies were indicated by following symbols: black arrow, karyopyknosis; white arrow, karyolysis; white triangle, cell swelling and disconnection; asterisk, coagulative necrotic debris. **b** Immunohistochemical staining of p-MLKL in tumor-adjacent epithelial (TAE) tissues of HNSCC patients. **c** H&E, p-RIP3, p-MLKL, NC staining on serial sections of HNSCC tumor tissues. Images were taken under ×50 and ×400 magnifications for each field. **d** P-MLKL-positive and p-MLKL-negative necrosis cluster and their corresponding H&E sections. **e** Immunohistochemistry analysis of MLKL expression in tumor and tumor-adjacent epithelial (TAE) tissues of HNSCC patients. f Comparison of MLKL expression in TAE and tumor tissues. Data are shown as mean ± SD, ***p value < 0.001(Mann–Whitney *U* test). **g** Western blotting analysis of the expression of necroptotic proteins in six pairs of patients’ tissues. **h** Kaplan–Meier survival analysis of the correlations between the overall survival (OS) and the level of MLKL or p-MLKL or tumor necrosis, respectively. The log-rank test was used to compare the survival rate between two groups, *p* value < 0.05 was considered significant. **i** Kaplan–Meier survival analysis of the correlations between the progression-free survival (PFS) and the level of p-MLKL or tumor necrosis, respectively. The log-rank test was used to compare the survival rate between two groups, *p* value < 0.05 was considered significant. Images were taken under ×100 and ×400 magnifications for each field unless stated otherwise.

To unveil the necroptotic status in HNSCC, we first assessed the expression of phospho-MLKL, which is currently the most recognized marker for necroptosis, in tumor and tumor-adjacent epithelial tissues (TAE) of HNSCC patients. P-MLKL can be detected in some tumor tissues, whereas no p-MLKL expression was detected in 40 stained TAE sections (Fig. 1a, b). P-MLKL-positive cells in tumor tissues mainly distributed in a clustered pattern. In comparison with the corresponding H&E sections it was observed that these p-MLKL-positive clusters exhibit clear necrotic morphologies, such as cell swelling, disconnection, karyopyknosis, karyolysis, etc. (Fig. 1a). In some case, the positive clusters exhibited typical coagulative necrosis features, with amorphous necrotic debris in the center and surrounded by necrotic cells (Fig. 1a). We then performed p-RIP3, p-MLKL, and H&E staining on serial sections of tumor tissues. We found the p-RIP3 was more widely stained than p-MLKL and not restrained to necrotic clusters. Enhanced p-RIP3-staining can be observed in p-MLKL-positive clusters suggests the activation of necroptotic pathway in these cells (Fig. 1c). Corresponding H&E sections also showed necrotic morphologies (Fig. 1c). Of note, no positive staining in the negative control (NC) group we set was observed confirming that the p-RIP3 and p-MLKL staining were not non-specific. These results further suggest that the necrosis traditionally observed in H&E sections could be necroptosis.

However, not all necrotic clusters observed in H&E sections were p-MLKL positive (Fig. 1d). Therefore, to assess the extent of necroptosis contribution in the tumor necrosis, we conducted H&E and p-MLKL staining on serial sections of 40 HSNCC tumor tissues. Numbers of necrotic clusters and coagulative necrosis in H&E sections and p-MLKL-positive clusters were counted (Fig. 1d). Results are shown in Table 1. Of the total 974 necrotic and coagulative necrosis, 545 were p-MLKL-positive, suggesting a necroptosis rate of 55.95%, and no statistical differences were observed between two staining patterns. Overall, these results clearly suggest that approximately half of the “necrotic foci” traditionally observed in HNSCC tissues can be owing to necroptosis.

**Table. 1:** Quantification results of p-MLKL and H&E-stained serial sections of 40 HNSCC patients.

Staining pattern	Staining method		Percentage	*χ*^*2*^	*P* value
	p-MLKL (+)	H&E			
Necrotic clusters	395	723	54.63%	0.553	0.457
Coagulative necrosis	150	251	59.76%		
Total	545	974	55.59%		

### Necroptosis is an independent prognostic factor in HNSCC

We then investigated whether necroptosis in HNSCC correlates with patients’ clinicopathological features. We included 191 HNSCC patients who received radical dissections between 2013 and 2018 (Table 2) and analyzed the expression of MLKL, p-MLKL in their tumor, and TAE tissues using immunohistochemistry (Fig. S1a, c). The extent of tumor necrosis was also analyzed using pathological H&E sections (Fig. S1b).

**Table. 2:** Patients’ clinicopathological characteristics and their correlation with MLKL expression.

Characteristics	MLKL expression				p-MLKL expression			Tumor necrosis		
	*N* = 191	Low	High	P*	Absent/focal	Extensive	P*	Absent/focal	Extensive	P*
		*n* = 96	*n* = 95		*n* = 130	*n* = 61		*n* = 100	*n* = 91	
*Gender*										
Male	129	68	61	0.328	83	46	0.112	61	68	**0.043**
Female	62	28	34		47	15		39	23	
*Age*										
<60	127	56	71	**0.016**	86	41	0.885	64	63	0.444
≥60	64	40	24		44	20		36	28	
*Tumor location*										
Tongue	111	58	53	0.227	70	41	0.671	55	56	0.292
Buccal	35	12	23		26	9		23	12	
Floor of mouth	14	7	7		10	4		9	5	
Gingiva	19	11	8		15	4		7	12	
Palate	8	4	4		6	2		4	4	
Oropharynx	3	3	0		1	0		1	0	
Others	1	1	0		2	1		1	2	
*Tobacco*										
No	112	55	57	0.704	77	35	0.808	60	52	0.689
Yes	79	41	38		53	26		40	39	
*Alcohol*										
No	145	64	81	**0.003**	96	49	0.329	74	71	0.516
Yes	46	32	14		34	12		26	20	
*Areca-nut*										
No	173	88	85	0.604	116	57	0.353	90	83	0.775
Yes	18	8	10		14	4		10	8	
*T stage*										
Tis/1/2	122	58	65	0.248	77	46	**0.029**	62	61	0.468
3/4	67	38	30		53	15		38	30	
*N stage*										
N0	99	54	45	0.219	64	35	0.293	50	49	0.595
N+	92	42	50		66	26		50	42	
*Clinical stage*										
0/I/II	76	44	32	0.086	47	29	0.134	38	38	0.596
III/IV	115	52	63		83	32		62	53	
*Histologic grade*										
Gx/G0	99	46	53	0.276	65	34	0.459	48	51	0.266
G1/G2	92	50	42		65	27		52	40	
*Recurrence*										
No	125	62	63	0.632	82	43	0.530	65	60	0.096
Yes	14	6	8		8	6		4	10	
*Lymph node metastasis*										
No	119	60	59	0.271	84	35	**<0.001**	64	55	**0.008**
Yes	19	7	12		5	14		4	15	
*Tumor progression*										
No	108	55	53	0.462	77	31	**0.004**	60	48	**0.013**
Yes	30	13	17		13	17		9	21	

^*^Chi-square test, *P* < 0.05 was considered statistically significant.

Bold value indicates *p* value < 0.05.

Results suggest that the MLKL expression is significantly upregulated in tumor tissues (Fig. 1e, f). Western blotting of six pairs of patients’ tumor-TAE tissues also shows a higher level of MLKL in tumor tissues, whesrea the RIP1 and RIP3 levels vary between different patients (Fig. 1g). Moreover, the MLKL expression are positively correlated with p-MLKL expression in tumor tissues (Table. S1), indicating that tumors with high MLKL expression may have a higher tendency to develop necroptosis. Correlation analyses show that high MLKL expression is correlated with the age under 60 years and alcohol consumption (Table 2). High p-MLKL expression is correlated with lower T stage, whereas high tumor necrotic level correlates with patients’ gender of male (Table 2). Importantly, both high level of p-MLKL and tumor necrosis correlate with lymph node metastasis and tumor progression (Table 2), indicating that necroptosis and necrosis may have potential influence on patient’s outcome.

Therefore, we further analyzed the relationship between the MLKL expression, p-MLKL expression, tumor necrosis, and the patients’ prognosis. Kaplan–Meier survival analyses shows that high level of all these three markers correlate with shorter overall survival (OS), whereas high level of p-MLKL and tumor necrosis also correlate with shorter progression-free survival (PFS, Fig. 1h). No correlation was observed between the MLKL expression and PFS and all these three markers does not correlate with recurrence-free survival (RFS, Fig. S1d). Univariate analysis showed that MLKL expression, p-MLKL expression, tumor necrosis, N stage, clinical stage, recurrence, cervical lymph node metastasis, and tumor progression are major risk factors for poor OS (Table 3), whereas p-MLKL expression, tumor necrosis, and areca-nut consumption are major risk factors for poor PFS (Table 4). Further multivariate analysis revealed that p-MLKL expression [HR (95%CI) = 4.500 (1.885, 10.743)], N stage [HR (95%CI) = 8.919 (3.471, 22.917)] and tumor progression [HR (95%CI) = 4.502 (2.016, 10.054)] were independent risk factors for patient’s OS (Table 3), and p-MLKL expression [HR (95%CI) = 3.277 (1.534, 7.001)] and areca-nut [HR (95%CI) = 2.376 (1.258, 4.490)] consumption are independent risk factors for PFS (Table 4).

**Table. 3:** Univariate and multivariate analysis of risk factors for patients’ overall survival (OS).

Variables	Univariate		Multivariate	
	*χ*^*2*^	*P* value	HR (95% CI)	*P* value
MLKL expression	7.300	**0.007**	—	0.214
p-MLKL expression	15.430	**<0.001**	4.500 (1.885, 10.743)	**0.001**
Tumor necrosis	4.815	**0.028**	—	0.105
N stage	16.769	**<0.001**	8.919 (3.471, 22.917)	**<0.001**
Clinical stage	11.978	**0.001**	—	0.552
Recurrence	13.479	**<0.001**	—	0.745
Lymph node metastasis	14.824	**<0.001**	—	0.592
Tumor progression	19.955	**<0.001**	4.502 (2.016, 10.054)	**<0.001**
T stage	2.315	0.128	—	—
Histologic grade	0.251	0.617	—	—
Tumor location	6.980	0.323	—	—
Gender	0.004	0.949	—	—
Age	0.009	0.926	—	—
Tobacco	0.594	0.441	—	—
Alcohol	1.510	0.219	—	—
Areca-nut	1.303	0.254	—	—

Bold value indicates *p* value < 0.05.

**Table. 4:** Univariate and multivariate analysis of risk factors for patients’ progression-free survival (PFS).

Variables	Univariate		Multivariate	
	*χ*^*2*^	*P* value	HR (95% CI)	*P* value
MLKL expression	1.144	0.285	—	—
p-MLKL expression	8.232	**0.004**	3.277 (1.534, 7.001)	**0.002**
Tumor necrosis	6.169	**0.013**	—	0.501
N stage	0.005	0.946	—	—
Clinical stage	0.203	0.653	—	—
T stage	0.623	0.430	—	—
Histologic grade	0.282	0.596	—	—
Tumor location	0.072	0.789	—	—
Gender	0.048	0.826	—	—
Age	0.002	0.962	—	—
Tobacco	0.331	0.565	—	—
Alcohol	1.555	0.212	—	—
Areca-nut	7.306	**0.007**	2.376 (1.258, 4.490)	**0.008**

Bold value indicates *p* value < 0.05.

Together, our results suggest that the extent of necroptosis can be an independent prognostic marker for OS and PFS of HNSCC patients. Meanwhile, it also implied that necroptosis may be a potential tumor-promoter in HNSCC.

### TSZ treatment induces non-apoptotic cell death in SCC25 and FaDu cells

**Fig. 2:**
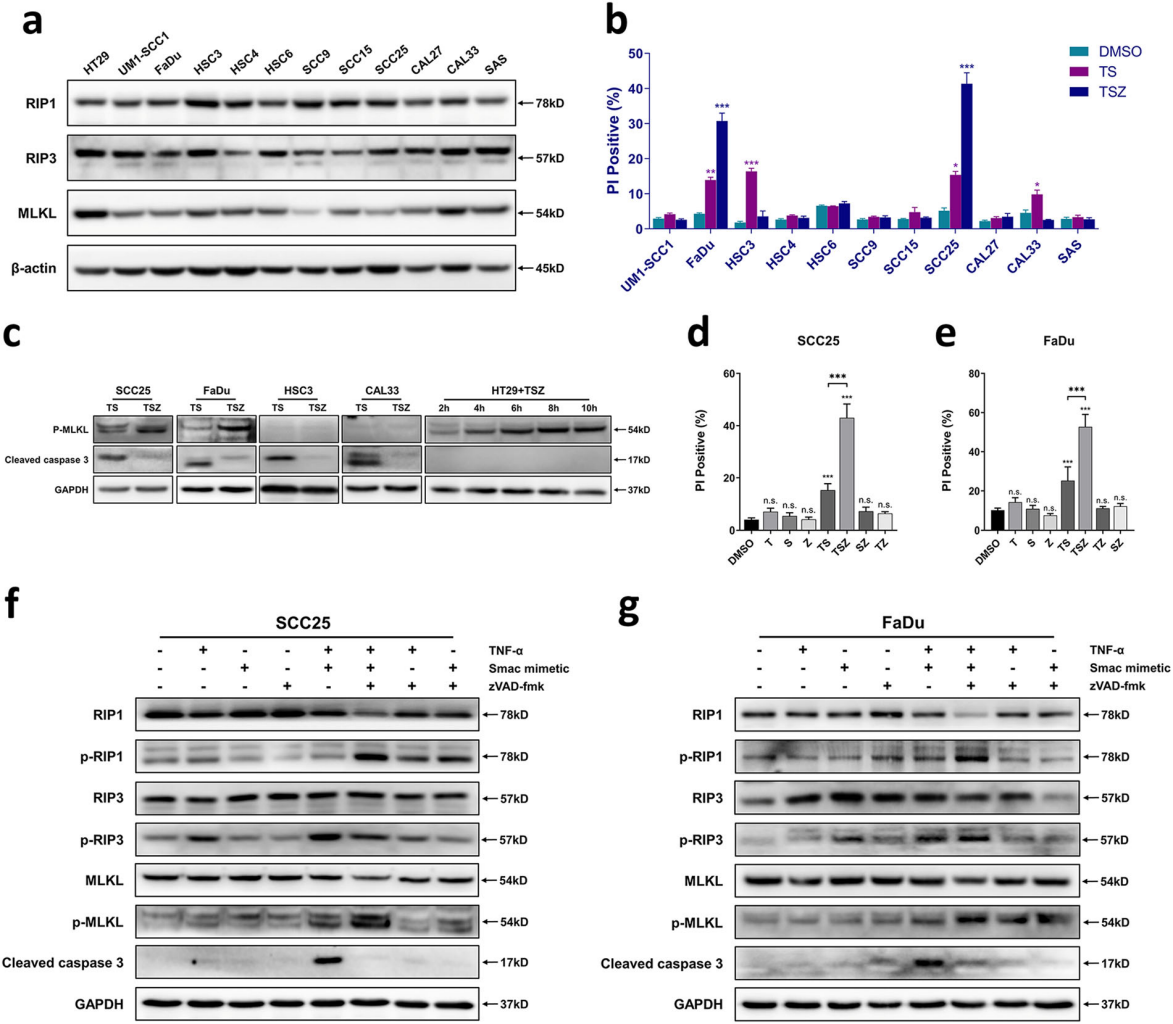
TSZ treatment induces non-apoptotic cell death in SCC25 and FaDu cells. **a** Western blotting-based screening of 11 HNSCC cell lines for the expression of necroptotic protein RIP1, RIP3, and MLKL. Human colorectal cancer cell line HT29 was used as a positive control. **b** 11 HNSCC cell lines were screened using TS or TSZ treatment for 24 hours. The cell death rate was determined using PI staining. DMSO was used as vehicle control. The indicated concentration was 30 ng/ml TNF-α, 1 µm Smac mimetic and 50 µm zVAD-fmk. c Western blot analysis of p-MLKL and cleaved caspase 3 expressions in SCC25, FaDu, HSC3, and CAL33 upon TS or TSZ treatment for 24 hours. HT29 cells treated by TSZ for 2–10 hours was used as a positive control for p-MLKL detection. **d** Cell death rate and **f** relative protein expression level of SCC25 after treatment of different combinations of 30 ng/ml TNF-α, 1 µm Smac mimetic, and 50 µm zVAD-fmk for 24 h. **e** Cell death rate and **g** relative protein expression level in FaDu cells after treatment with different combinations of 30 ng/ml TNF-α, 1 µm Smac mimetic, and 100 µm zVAD-fmk for 48 h. Identical concentrations of T, S, Z, and treated-time were used in subsequent experiments unless otherwise stated. Data are shown as mean ± SD of three independent experiments. *p value < 0.05, **p value < 0.01, ***p value < 0.001 (one-way ANOVA and Turkey’s multiple comparisons test).

Next, we sought to induce necroptosis in HNSCC cell lines to construct a cellular model of necroptosis. We first screened 11 HNSCC cell lines using western blotting and found that they all expressed different levels of RIP1, RIP3, and MLKL (Fig. 2a), which are the core components of the necroptotic pathway. Based upon the classic necroptotic pathway and extrinsic apoptotic pathway, we screened these 11 cell lines with combined treatment of TNFα + Smac mimetic (TS) or TNFα + Smac mimetic+zVAD-fmk (TSZ). Results suggest that HSC3 and CAL33 were only sensitive to TS treatment, whereas SCC25 and FaDu were sensitive to both TS and TSZ-induced cell death (Fig. 2b). Adding a pan-caspase inhibitor zVAD-fmk to TS treatment (TSZ) resulted in elevation rather than inhibition of cell death in SCC25 and FaDu cells (Fig. 2c), indicating that TSZ could induce non-apoptotic cell death in these two cell lines. Western blot analysis further revealed that apoptotic marker cleaved caspase 3 can be detected in all these four cell lines under the TS treatment (Fig. 2c), suggesting the cell death in TS group was only apoptosis. The necroptotic marker p-MLKL can only be detected only in SCC25 and FaDu under TSZ treatment along with absence of cleaved caspase 3 (Fig. 2c). On comparison of results with positive control HT29 (Fig. 2c) we can infer that TSZ-induced cell death was potentially necroptosis.

### TSZ-induced cell death is necroptosis

Further to investigate along the similar lines, we treated SCC25 and FaDu cells with different combination of T, S, Z. Findings suggest that only TS and TSZ treatment can induce significant cell death (Fig. 2d, e). Western blotting further revealed that the phosphorylation level of RIP1, RIP3, and MLKL has significantly increased in TSZ group (Fig. 2f, g), suggesting activation of necroptotic pathway.

**Fig. 3:**
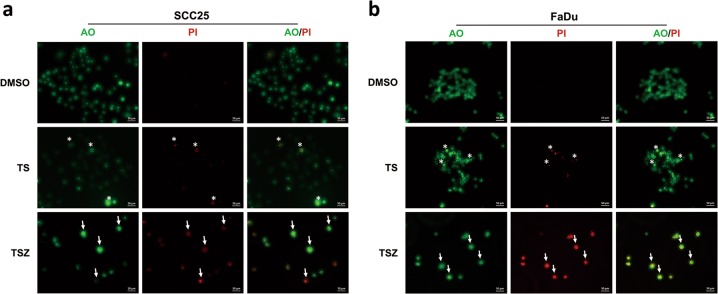
TSZ induced cell death shows necrotic features. AO-PI double-fluorescent staining of **a** SCC25 and **b** FaDu treated with DMSO, TS, and TSZ, respectively. Typical apoptotic cells (strong, lump-like AO staining, late stage PI staining) are indicated with an asterisk. Typical necrotic cells (early PI staining, AO staining showing intact nuclear, lack of lump-like AO staining) are indicated with an arrow.

**Fig. 4:**
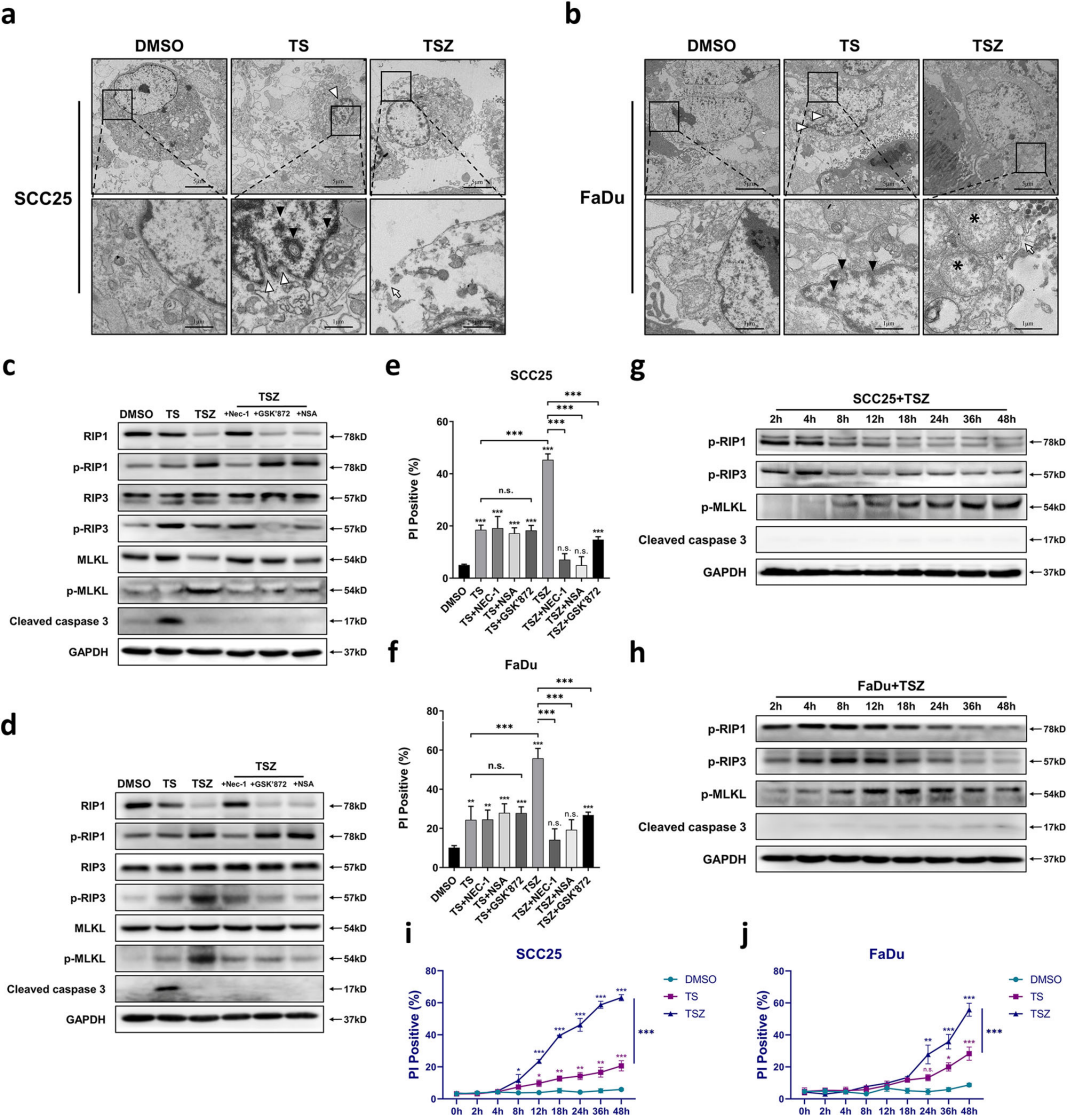
TSZ-induced non-apoptotic cell death is necroptosis. Transmission electron microscopy (TEM) analysis of the ultrastructure of **a** SCC25 b FaDu cells under DMSO, TS, and TSZ treatment. Morphological changes are indicated by following symbols, white triangle: nuclear shrinkage; black triangle: chromatin condensation and margination; white arrow: discontinuous membrane; black asterisk: swollen mitochondria. Relative protein expression **c** and the rate of cell death **e** of SCC25 treated with TSZ supplemented with indicated concentrations of Nec-1, GSK’872, and NSA, respectively. Relative protein expression **d** and cell death rate **f** of FaDu cells treated with TSZ supplemented with indicated concentrations of Nec-1, GSK’872, and NSA, respectively. Continuous detection of the phosphorylation of RIP1, RIP3, MLKL upon TSZ treatment in **g** SCC25 and **h** FaDu at different time points ranging from 2 to 48 hours. Continuously detected cell death rate between 0 and 48 hours in **i** SCC25 and **j** FaDu upon DMSO, TS, TSZ treatment. Data are shown as mean ± SD of three independent experiments. *p value < 0.05, **p value < 0.01, ***p value < 0.001 (one-way ANOVA and Turkey’s multiple comparisons test).

AO-PI double staining detected strong lump-like AO staining in the TS group, which can be interpreted as condensed chromatin or nuclear debris. PI staining was found only in few cells with obvious abnormal morphology, suggesting enhanced permeability of plasma membranes in the late stage of cell death (Fig. 3). These features further reflect apoptotic morphologies of TS-treated cells. Conversely, TSZ-treated cells showed typical necrotic features (Fig. 3) such as early-stage membrane permeabilization (increased PI staining in cells with relatively normal morphology), intact nucleus and no chromatin condensation (lack of lump-like AO staining). Quantification confirms that the cell death in TS group are mainly apoptosis while TSZ-treated cells died predominantly in a necrotic-like manner (Fig. S2a). Similarly, transmission electron microscopy (TEM) also demonstrated typical apoptotic morphology in the TS group, including chromatin condensation and margination, shrinkage of cell and nuclear membranes, while maintaining the integrity (Fig. 4a, b). In contrast the TSZ group showed disruption of membrane continuity, chromatin rupture, and swelling of organelles, which are classical markers for necrosis (Fig. 4a, b). Continuous imaging (Movie. S1, S2) also revealed that cells treated with TSZ exhibited typical necrotic changes (cell disconnection and swollen, nuclear remain intact), whereas TS-treated cells showed apoptotic morphology (cell and nuclear shrinkage, apoptotic-body formation).

Next, we used three small-molecule inhibitors, Necrostatin-1, GSK’872, necrosulfonamide (NSA) targeting RIP1, RIP3, and MLKL, respectively. Findings suggest that they all can significantly inhibit TSZ-induced cell death without having any effect on the TS group (Fig. 4e, f). Western Blot analysis showed that the addition of Necrostatin-1, GSK’872, and NSA can inhibit the phosphorylation of RIP1, RIP3, and MLKL, respectively, and ultimately reduce the level of p-MLKL (Fig. 4c, d). Furthermore, we continuously detected the phosphorylation level of necroptotic proteins at different time points within 2–48 hours of TSZ treatment. We also observed sequential phosphorylation of RIP1, RIP3, and MLKL after TSZ induction (Fig. 4g, h) along with a gradual increase in cell death rate (Fig. 4i, j). This further confirmed the necroptosis pathway was responsible for TSZ-induced cell death in SCC25 and FaDu cells.

Overall, our results provided evidence in support of the fact that TSZ-induced cell death was necroptosis, whereas TS-induced cell death is apoptosis in SCC25 and FaDu cells.

### The expression level of MLKL affects the sensitivity to TSZ treatment of HNSCC cells

**Fig. 5:**
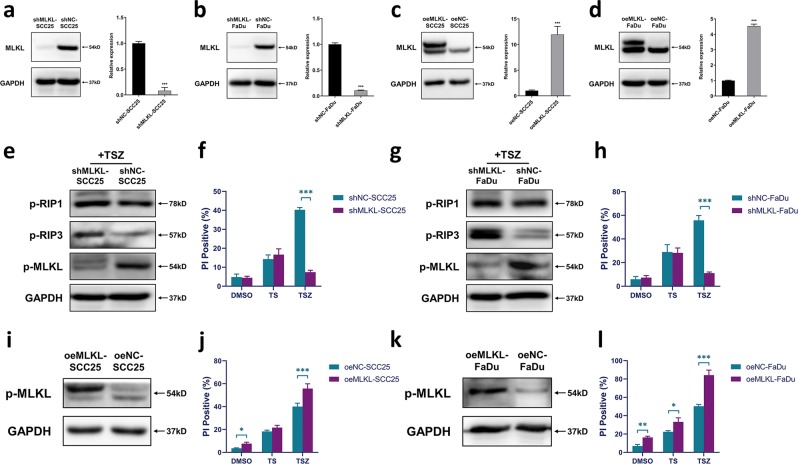
MLKL level affects the sensitivity to TSZ treatment in HNSCC cells. Validation of stable MLKL knockdown in **a** SCC25 and **b** FaDu using western blotting and qRT-PCR. Validation of stable MLKL overexpression in **c** SCC25 and **d** FaDu using western blotting and qRT-PCR. Western blot analysis of protein phosphorylation in **e** shMLKL-SCC25, **g** shMLKL-FaDu, **i** oeMLKL-SCC25, and **k** oeMLKL-FaDu upon TSZ treatment. Cell death rate of **f** shMLKL-SCC25, **h** shMLKL-FaDu, **j** oeMLKL-SCC25, and **l** oeMLKL-FaDu under DMSO, TS, TSZ treatment. Data are shown as mean ± SD of three independent experiments. *p value < 0.05, **p value < 0.01, ***p value < 0.001 (one-way ANOVA and Turkey’s multiple comparisons test).

MLKL is the terminal executor of necroptosis and is considered as the most specific molecule involved in the regulation of the process^11^. Therefore, in the next step we evaluated the process of MLKL-mediated response regulation to TSZ treatment in HNSCC cells. We constructed and validated overexpression and knockdown-MLKL stable cell lines in SCC25 and FaDu (Fig. 5a–d). The knockdown of MLKL markedly inhibited the TSZ-induced necroptosis but did not affect the TS-induced apoptosis (Fig. 5f, h). Western blot analyses demonstrate that MLKL-knockdown results in significantly reduced phosphorylation of MLKL (Fig. 5e, g), which also provided additional evidence of TSZ-induced cell death being necroptosis. Of note, higher level of p-RIP3 and a slightly higher level of p-RIP1 are observed in shMLKL groups, which probably be a consequential accumulation owing to the downregulated MLKL phosphorylation. To confirm this, we conducted the shMLKL-knockdown assay in HT29 cells and observed both p-RIP1 and p-RIP3 were higher in shMLKL group. (Fig. S2b). However, this phenomenon was absent in previous experiment when NSA was used to inhibit the phosphorylation of MLKL. Therefore, another possibility is the knockdown of MLKL results in an abnormal activation of RIP1 and RIP3. Further research is needed to fully address the underlying mechanisms of this phenomenon.

In contrast, MLKL overexpression resulted in increased phosphorylation and cell death after TSZ treatment (Fig. 5i–l). Taken together, our results suggest that the expression level of MLKL can affect the sensitivity of cells to TSZ-induced necroptosis. Interestingly, we also noticed that dimethyl sulfoxide (DMSO) and TS treatment can induce a slightly higher cell death rate in MLKL-overexpressing cell lines (Fig. 5j, l). We had also observed that untreated MLKL-overexpression cell lines exhibit higher death rates as compared to their original cell lines (data not shown). The increase of this “spontaneous” cell death upon MLKL overexpression implies that elevated MLKL expression may confer HNSCC cells a higher propensity to develop necroptosis, which is in consistent with our result that the p-MLKL expression is correlated with MLKL expression in patients’ tissues (Table. S1).

### Necroptotic cells promote the migration and invasion of HNSCC cells in vitro through releasing DAMPs

**Fig. 6:**
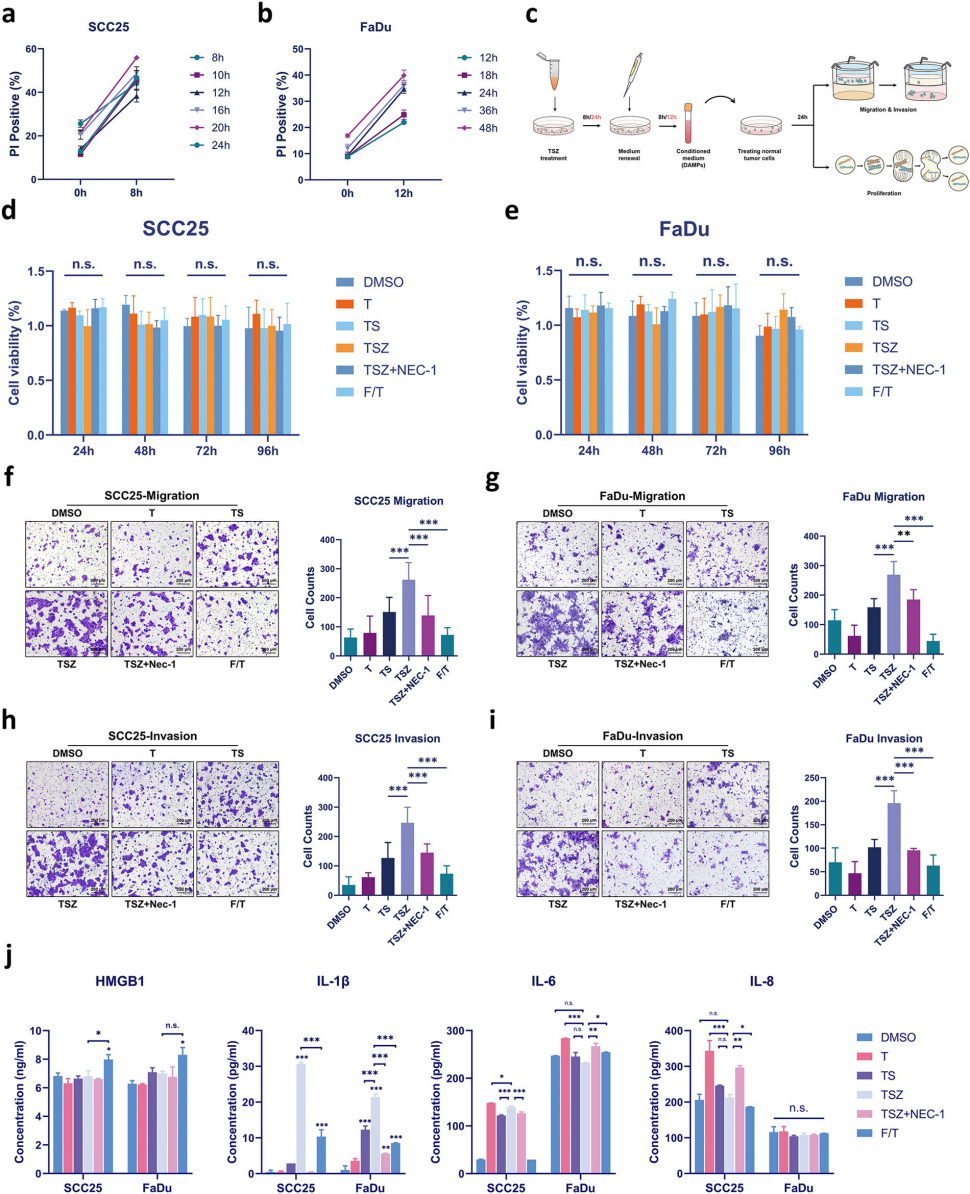
Necroptotic DAMPs promote tumor cell migration and invasion in SCC25 and FaDu. SCC25 and FaDu were induced with TSZ for different time and then the culture medium was renewed. The cell death rate of **a** SCC25 and **b** FaDu before the medium renewal (0 h) and 8/12 hours after (8 h/12 h) was measured. **c** Flow chart of the collection of DAMPs and further analysis on its potential impact. The proliferation rate of **d** SCC25, and **e** FaDu treated with different conditioned medium for 24, 48, 72, and 96 hours. Transwell migration and invasion assay of **f**, **h** SCC25 and **g**, **i** FaDu cells treated with different conditioned medium for 24hours. **j** Concentrations of HMGB1, IL-1β, IL6, and IL8 in the supernatant of treated SCC25 and FaDu cells. Data are shown as mean ± SD of three independent experiments. *p value< 0.05, ** *p* value< 0.01, *** *p* value< 0.001 (one-way ANOVA and Turkey’s multiple comparisons test).

Our results have indicated that necroptosis might be a potential tumor-promoter in HNSCC. Since releasing of DAMPs is one of the key features of necroptosis, we then investigated whether it contributes to the tumor progression of HNSCC cells. We first treated SCC25 and FaDu with TSZ for different time and then renewed the culture medium to eliminate the stimulation of drugs. Interestingly, we observed that cell death rate continued to increase after the medium renewal (Fig. 6a, b). Among different time points, 8 hours and 24 hours’ TSZ treatment resulted in the highest increase of cell death rate in SCC25 and FaDu, respectively (Fig. 6a, b). Based on this phenomenon, we designed an experimental routine for collecting DAMPs and analyzing its potential impact on normal HNSCC cells (Fig. 6c). Results show that comparing with apoptotic (TS) and accidental necrotic DAMPs (F/T), the necroptotic DAMPs (TSZ) from SCC25 and FaDu can not only promote the migration and invasion of normal SCC25 and FaDu cells (Fig. 6f–i), but also promote the migration and invasion of TSZ-insensitive HSC3 and HSC6 cells (Fig. S2d, e). Meanwhile, the necroptotic DAMPs do not affect the proliferation rate of HNSCC cells (Fig. 6d, e). However, the necroptotic DAMPs released by HT29 does not show similar effect on the migration and invasion of SCC25 cells (Fig. S2f), suggesting this tumor-promoting ability of necroptotic DAMPs might be affected by inter-tumoral heterogeneity. Moreover, upon the inhibition of necroptotic cell death through addition of Nec-1 or knockdown of MLKL, the conditioned medium failed to affect the migration and invasion of treated cells (Fig. 6f–I, Fig. S2c), indicating that the tumor-promoting effect was cell death dependent rather than drug dependent. Our results strongly suggest that DAMPs release in necroptosis is not simply passive leakage of intracellular components. Instead, it might involve multiple other intracellular pathways and contain certain components that are absent in the DAMPs of apoptosis and accidental necrosis.

To test this hypothesis, we detected the releasing of several DAMPs that may involve in the regulation of tumor cell migration and invasion. We found the releasing of classic DAMPs, HMGB1 is significantly higher in F/T group, suggesting that necroptosis will not result in increased leakage of intracellular components (Fig. 6j). This also provide evidence that the freeze–thaw treatment well imitated the membrane rupture and DAMPs release of accidental necrosis. And necroptotic cells does not release more IL6 and IL8 than other groups as well (Fig. 6j). However, the releasing of IL-1β is significantly higher in TSZ group than TS and F/T groups (Fig. 6j). And the inhibition of IL-1β releasing by the addition of Nec-1 clearly shows the necroptotic-death dependency of IL-1β releasing (Fig. 6j). These results supported the hypothesis that the necroptotic DAMPs are released rather in a more active manner and may contain some unique components comparing to apoptosis and accidental necrosis.

Taken together, our findings suggest that necroptotic cells can promote the migration and invasion of normal HNSCC cells in vitro through releasing DAMPs. And IL-1β is one of the necroptotic-specific DAMPs that may potentially involve in the regulation of cell migration an invasion.

## Methods and materials

### Patients and tissue samples

A total of 191 HNSCCs patients undergone a radical surgery between 2013 to 2018 at the Department of Oral and Maxillofacial Surgery, Hospital of Stomatology, Sun Yat-sen University, were included in this study. No chemotherapy or radiotherapy were performed before the surgery on all the patients. Tumor and adjacent epithelial tissues were collected and fixed in 4% paraformaldehyde, embedded in paraffin, and used for HE and immunohistochemistry. Among them, 40 patients’ tumor and TAE samples that were continuously included from September to December in year 2018 were used for serial sectioning and H&E and p-MLKL staining. Clinicopathological parameters and follow-up data for all the study participants were collected. The starting point for patients’ survival was the date of surgery. The endpoint for OS, RFS, and PFS was patient death, locoregional recurrence, and tumor progression (locoregional recurrence or lymph node/distant metastasis), respectively. Tumor staging and histological grading were based upon the 8th edition of UICC/AJCC TNM classification. The study was approved by the Ethics Committee of the Stomatological Hospital of Sun Yat-sen University (Approval number: ERC- [2017]-29). Informed consent was obtained from all the study participants.

### Immunohistochemistry

The tissue sections were heated for 2 hours at 60°C followed by deparaffinization and rehydration. The antigen was retrieved under high pressure for 3 minutes in citrate buffer (pH 6.0) and gently cooled down to room temperature. After blocking the endogenous peroxidases, the sections were incubated with primary antibody at 4°C overnight and then with secondary antibody HRP-conjugated anti-goat IgG (Gene Tech, Shanghai, China) for 1 h at room temperature. The DAB chromogenic kit (Gene Tech, Shanghai, China) was used for staining the targeting proteins and hematoxylin was used for nuclear staining. Stained sections were sealed with neutral resin after dehydration and captured automatically using Aperio Digital Pathology Slide Scanners (Leica, Germany).

The scoring of MLKL expression was conducted following a previously described method^17,18^. In brief, the extent was scored as the percentage of the cells that showed cytoplasmic and/or membranous staining: 0 = < 1%, 1 = 1–10 %, 2 = 11–50%, 3 = 51–80%, and 4 = > 80%. The intensity was scored as 1 = weak, 2 = moderate, and 3 = strong. Scoring was performed by two pathologists without prior knowledge of the clinical follow-up data for each case. The overall score is then calculated as (1 + intensity/3) * extent. For each section, 5 HPF (×200) was analyzed and the mean was calculated as the final score. The expression level was defined as none=0, low=0–2.667, medium=2.667–5, and high=5–8. Then, it was further binarized as: low=0–5, High=5–8, for correlation and survival analysis. The representative images of different scores were shown in Fig. S1c.

The scoring of p-MLKL and tumor necrosis were conducted following a previously described method^19^. In brief, the p-MLKL expression/tumor necrosis was scored as the percentage of positive cells/necrosis: 0, no positive staining/necrosis; 1, <10% positive staining/necrosis; 2, 10–30% positive staining/necrosis; and 3, >30% positive staining/necrosis. The scoring was than binarized as Absent/Focal (score=0–2) and extensive (score=3) for correlation and survival analysis. The representative images of different scores were shown in Fig. S1a, b.

### Determining the contribution of necroptosis to tumor necrosis in HNSCC

Immunohistochemical staining of p-MLKL and H&E staining on serial-sectioned tumor tissues of 40 patients were performed. Five fields under ×100 magnification were randomly selected and counted in each H&E section. Necrosis clusters and coagulative necrosis were counted separately. A necrosis cluster is defined as a cluster of cells that exhibits clear necrotic morphology (Fig. 1A) and can be separated from adjacent normal tumor cells, tumor-infiltrated lymphocytes (TILs) and tumor stroma. A coagulative necrosis is defined as a cluster of lighter-stained necrotic debris containing no cellular microstructures while the tissue contour partially remained (Fig. 1A). The corresponding fields on p-MLKL sections were analyzed and a cluster was defined as p-MLKL-positive if over 50% of the cells were stained. The counting was performed by two pathologists without the former knowledge of patients clinicopathological characteristics.

### Cell culture and reagents

HNSCC cell lines SCC9 (ATCC Cat# CRL-1629, RRID: CVCL_1685), SCC25 (ATCC Cat# CRL-1628, RRID: CVCL_1682), Cal27 (ATCC Cat# CRL-2095, RRID: CVCL_1107), SCC15 (ATCC Cat# CRL-1623, RRID: CVCL_1681) were purchased from American Type Culture Collection (ATCC). SAS (JCRB Cat# JCRB0260, RRID: CVCL_1675) was purchased from JCRB cell bank. FaDu (ATCC Cat# HTB-43, RRID: CVCL_1218) was generously gifted by Dr. Su Yuxiong (Hong Kong University). UM-SCC1 (Millipore Cat# SCC070, RRID: CVCL_7707), HSC3 (TKG Cat# TKG 0484, RRID: CVCL_1288), HSC4 (JCRB Cat# JCRB0624, RRID: CVCL_1289), HSC6 (RRID:CVCL_A615), CAL33 (DSMZ Cat# ACC-447, RRID:CVCL_1108), and colorectal adenocarcinoma cell line HT29 (ATCC Cat# HTB-38, RRID: CVCL_0320) were preserved in Guangdong Province Key Laboratory of Stomatology. SCC25, SCC15, SCC9, SAS, UM-SCC1 were cultured in DMEM/F12 1:1 (Gibco, USA) supplemented with 10% fetal bovine serum (FBS, Gibco, USA) and 400 ng/ml hydrocortisone (Solarbio, Beijing, China). FaDu was cultured in MEM (Gibco, USA) supplemented with 10% FBS. Other cell lines were cultured in DMEM (Gibco, USA) supplemented with 10% FBS.

Human recombinant TNF-α was purchased from PeproTech (USA). Smac mimetic AT-406 (SM-406), pan-caspase inhibitor zVAD-fmk, RIP1 inhibitor Necrostain-1 (Nec-1), RIP3 inhibitor GSK’872, and MLKL inhibitor NSA were purchased from Selleck (Selleckchem, Houston, TX, USA). Antibodies against RIP1 (Cell Signaling Technology Cat# 3493, RRID: AB_2305314), p-RIP1 (Cell Signaling Technology Cat# 44590, RRID: AB_2799268), RIP3 (Cell Signaling Technology Cat# 13526, RRID: AB_2687467), p-RIP3 (Cell Signaling Technology Cat# 93654, RRID: AB_2800206), cleaved caspase 3 (Cell Signaling Technology Cat# 9661, RRID: AB_2341188) and β-actin (Cell Signaling Technology Cat# 12620, RRID:AB_2797972) were purchased from Cell Signaling Technology (USA). Anti-MLKL (Abcam Cat# ab184718, RRID: AB_2755030) and anti-phospho-MLKL (Abcam Cat# ab187091, RRID: AB_2619685) were purchased from Abcam (England). Anti-GAPDH (CWBio Cat# CW0100M, RRID: AB_2801390) antibody was purchased from CWBIO (China).

### Induction/inhibition of necroptosis

Combined treatment of TNF-α, Smac mimetic, and zVAD-fmk was used to induce necroptosis in HNSCC cell lines. Specifically, cells were pretreated with zVAD-fmk and Smac mimetic for 1–4 hours depending upon the cell lines. Then 30 µg/ml TNF-α was added into the culture medium and treated for indicated time. Cells treated with the corresponding volume of DMSO was used as vehicle control. For inhibition of necroptosis several inhibitors such as Nec-1 (10 µm), GSK’872 (5 µm), NSA (1 µm) were added together with S and Z before stimulation with TNF-α. The colorectal adenocarcinoma cell line HT29 was used as positive control.

### Flow cytometry

Propidium iodide (PI, BD Biosciences, USA) staining and flow cytometry (Beckman Cytoflex, Beckman Coulter, USA) was used for analysis of cell death. Cells receiving different treatments were harvested, washed twice with pre-cooled PBS and resuspended in 100 µl binding buffer (BD Biosciences, USA). Five microliter of PI solution was added to each tube and incubated for 5 minutes in dark at room temperature. After the incubation, 400 µl binding buffer was added to each tube and flow cytometry was performed. The percentage of PI-positive cells was considered as the rate of cell death. Three duplications are set for each group in one experiment.

### Western blotting

Cells were harvested and lysed in radioimmunoprecipitation buffer (Beyotime, China). Quantification was performed using the BCA protein assay kit (CWBIO, China). Proteins were separated by 10% SDS-PAGE gel electrophoresis and transferred to polyvinylidene fluoride (PVDF) membranes (Millipore, USA), after blocking with 2% skim milk or bovine serum albumin (BSA). The membrane was then treated with primary antibodies at 4°C overnight and then washed using 1× Tris-Bufferd Saline and Tween 20 (TBST, CWBIO, China). In the end the membranes were incubated with secondary antibodies (EMAR, China) for 1 h at room temperature. The protein expression level was measured by Immobilon ECL Ultra Western HRP Substrate (Millipore, USA) using GeneGnome XRQ system (Syngene, USA).

### Gene overexpression and knockdown

Overexpression MLKL plasmid was custom synthesized from Hanbio Technology (Shanghai, China). shMLKL plasmid (psi-LVRU6P-MLKL) was purchased from GeneCopoeia, USA (target sequence: CACCTGAACTCCACGGAAA). Plasmid packaging and transfection were based on Lenti-Pac FIV Expression Packing Kit (GeneCopoeia, USA). Lentiviruses were then harvested and used for infecting SCC25 and FaDu cells for 48 h. In all, 1–2 µg/ml puromycin was added into the culture medium to screen out stably transfected cells. Transfection efficiency was measured by western blotting and RT-PCR.

### Quantitative real-time PCR (qRT-PCR)

Total RNA was extracted using RNAzol (Molecular Research Center, Inc, USA) agent. It was dissolved in DNase/RNase-free water (Ribobio, Guangzhou, China) and quantified using NanoDrop2000. RNA reverse transcription process was performed following the manufacturer’s instructions (PrimeScript RT reagent Kit, Takara, Japan). RT-PCR was performed using SYBR PrimeScript RT-PCR Kit (Roche, USA) following the manufacturer’s protocol. Each group was duplicated three times in one experiment.

Primer sequences:

MLKL: Forward, 5′- CAACCTGAAGTAACAGCGAGA-3′

Reverse, 3′-GGCTAATGGGGAGATAGAAAA-5′

GAPDH: Forward, 5′-CCCTGTTGCTGTAGCCAAATT-3′

Reverse, 3′-CACCCACTCCTCTACCTTCGA-5′

### TEM

Adherent cells under different treatments were scraped and centrifuged into a dense cell cluster and fixed using 2.8% glutaraldehyde for at least 24 h at 4°C. The cell clusters were made into ultra-thin sections and finally photographed using TEM (JEM-1400 electron microscope, JEOL Ltd, Japan) to detect morphological changes.

### Fluorescent staining

AO-PI double staining was used to differentiate between apoptosis and necrosis-like cell death. This staining process is consisting of two nucleic acid dyes Acridine Orange (AO, Solarbio, Beijing China) and PI (Solarbio, Beijing, China). AO possesses membrane permeability, whereas PI can only enter cells with ruptured membrane. In brief, the treated cells were washed twice with PBS. AO and PI were diluted in PBS to a final concentration of 5 µg/ml and 50 µm, respectively. Cells were incubated in dark with the dye solution cells at 37 °C for 20 minutes. After gently washing twice with PBS, cells were visualized under a fluorescent microscope (Axio, ZEISS, Germany). For quantification, five HPF (×200) were selected for each group and the number of normal, apoptotic, and necroptotic cells were calculated. The Chi-square test was used to determine the statistical differences between each group.

### Collection of DAMPs

After inducing SCC25 and FaDu with different treatments for 8 and 24 hours, respectively, culture medium was renewed to eliminate the stimulation of drugs. Cells were cultured for additional 8 or 12 hours and the culture medium were collected. The conditioned medium was centrifuged under 2000 × *g* for 10 minutes to eliminate cell debris before using for further experiments. For collection of the DAMPs from accidental necrosis, cells were cultured for the same time and went through three times frozen-thaw (F/T) cycles^20–22^ and centrifuge to eliminate cell debris.

### Cell proliferation assay

Cells suspended in 200 μl of culture medium were seeded into 96-well plate and cultured for 24 hours. Then the cells were treated with different conditioned medium for 24, 48, 72, and 96 hours, respectively. The proliferation rate was measured following the manufacture’s instruction of Cell Counting kit-8 (CCK-8, Dojindo, Japan). Three duplications are set for each group in one experiment.

### Transwell migration and invasion assay

In all, 6.5-mm-diameter polycarbonate filters (8-μm pore size) and 24-well plates were used. Cells were treated with different conditioned medium for 24 hours, and then resuspended in 200 μl serum-free MEM or DMEM/F12 (1:1) and seeded in the top chamber and the bottom chamber was filled with 800 μl MEM or DMEM/F12 (1:1) containing 10% FBS. Cells were allowed to migrate for 24 hours. For invasion assay, the filter was pre-coated with 1:20 diluted Matrigel (BD biosciences) for 2 hours, and then cells were seeded and cultured as the same with migration assay. The cells were fixed with 4% paraformaldehyde and stained by crystal violet. Five images per chamber were taken using inverted microscope under ×100 magnification (Axio, ZEISS, Germany) and the migrated cells in lower chamber were counted using ImageJ software.

### ELISA assay

SCC25 and FaDu cells were seeded into six-well plate and treated for 24 and 48 hours, respectively. The supernatants were collected and centrifuged under 2000 × *g* for 10 minutes to eliminate cell debris. Human IL-1β ELISA kit, Human IL6 ELISA kit, Human IL8 ELISA kit (NeoBioscience, Guangdong, China), and Human HMGB1 ELISA kit (Telenbiotech, Guangzhou, China) were used to detect the concentration of indicated molecules following the manufacture’s instruction.

### Statistical analysis

SPSS 20.0 software (SPSS, USA) was used for statistical analysis. Mann–Whitney *U* test was used to compare the difference of MLKL expression between tumor tissues and TAE tissues. Kaplan–Meier log-rank survival analysis was performed to determine the prognostic factors for OS, RFS, and PFS. Multivariate Cox-regression was used to determine the independent prognostic factors. A chi-square test was used to determine the differences in the clinicopathological features between two expression groups of patients. The unpaired student’s *t* test was used for the comparison of two groups of quantitative data (cell death rate, cell viability, mRNA relative expression, cell counts, and cytokine concentration). One-way ANOVA was used for comparing multiple groups of quantitative data, Turkey’s multiple comparisons test was used for pairwise comparison between each group. *p* value < 0.05 was considered statistically significant. Quantitative data are showed as mean ± SD unless stated otherwise. All experiments are repeated at least twice in this research.

## Discussion

In this study, we unveiled the necroptosis status in HNSCC for the first time. Necrosis is a common pathological phenomenon in HNSCC^23^. It has been reported to correlate well with invasive phenotypes and poor prognosis in multiple cancers, including breast cancer, lung cancer, colorectal cancer, and other tumor types^16,19,24–29^. These researchers deemed that degree of necrosis were directly related with hypoxia level inside the tumor, which was actually responsible for the invasive phenotype and tumor progression. Our study demonstrated that around half of the necrosis in HNSCC can be attributed to necroptosis. Corroborating these results Jiao et al.^30^ also reported that p-MLKL expression can be detected around the necrotic foci in tissue samples of mouse MMVT-PyMT breast cancer and human breast, lung, and liver cancer. Our findings provided a novel insight that the “necrotic foci” observed in pathological sections may include more complex components such as necroptosis and pyroptosis, rather than simple passive necrosis that caused by factors such as hypoxia, ischemia, and nutrient deprivation. Moreover, although both p-MLKL expression and tumor necrosis were correlated with OS and PFS in univariate analysis, the necrosis was later excluded in the multivariate cox-regression model. This partially consisted with previous research that tumor necrosis did not correlate with local-regional control of HNSCC^23^. It also indicated that the extent of necroptosis might be a more specific prognostic marker than necrosis.

TNFα-TNFR is a classic pair of cell death ligand-receptor. Stimulation of TNFα exclusively can activate the classic NF-κB pathway, which further mediates cell survival and proliferation. Smac mimetic inhibits cIAPs’ activity and disturbs the ability of TNFα-stimulated cells to form a stable complex I, instead it promotes complex II formation, which leads to apoptosis. Under normal conditions, caspase 8 activity can initiate apoptosis and inhibit the occurrence of necroptosis^31^. Therefore, the combined use of broad-spectrum caspase inhibitor zVAD-fmk with TNFα and Smac mimetic can induce necroptosis. TSZ-induced HT29 is the earliest and most commonly used cellular model for studying necroptosis. Wang et al.^32–34^ initially used this model in a series of studies and unveiled the classic necroptotic RIP1/RIP3/MLKL pathways. Therefore, TSZ-treated HT29 was used as a positive control in our study. The two most significant features of necroptosis are necroptotic pathway regulation and necrotic-cell morphology^11,12^. However, except for HT29 cell line the validation of necroptosis in existing studies failed to meet both of the criteria^35–37^. Obviously, solely detection of either pathway activation or morphological changes can easily lead to false positive result. Based upon the existing evidence, we believe that p-MLKL, which is a currently well recognized marker of necroptosis^11^, along with necrotic-like morphological changes, can be two key markers in validating necroptosis. Eytan et al.^9^ had observed in their study that TNF-α plus Smac mimetic Birinapant can induce non-apoptotic cell death in head and neck cancer cell line UM-SCC-46, and Necrostatin-1 can rescue the cell death. However, further evidence that supported the cell death to be necroptosis were absent. In addition, RIP1 had been demonstrated as an essential molecule in the regulation of apoptosis and several researchers had reported that inhibition of its kinase activity can rescue cells from apoptosis (though we did not observe this phenomenon in our model)^38–41^. This again supported our notion that both pathway activation and morphological changes should be validated to conclude a cell death is necroptosis. In the present study, we focused on these two points and thoroughly verified that TSZ-induced non-apoptotic cell death is necroptosis, hence provided a well-characterized cellular model for future research on necroptosis in HNSCC.

We also found that most of the screened HNSCC cell lines were insensitive to TSZ-induced cell death corroborating with the findings of Hannes et al.^35^ in pancreatic cancer. Najafov et al.^42^ screened 941 tumor cell lines and found that 780 of them were insensitive to TSZ treatment. In contrast, we found that the HNSCC cell lines FaDu and SCC25 are sensitive to TSZ treatment, whereas HSC3 is not sensitive. Najafov et al.^42^ further identified that AXL overexpression and BRAF mutation could be responsible for RIP3 expression loss during cancer development. However, Jiao et al.^30^ found the opposite phenomenon in mouse MMVT-PyMT breast cancer model and suggested that the expression of RIP3 was lower in early stage while it significantly increased in advanced tumors. In our screening of HNSCC cell lines, we observed that every cell line expressed different levels of RIP1, RIP3, and MLKL, indicating that the insensitivity to TSZ treatment is not due to the expression level of necroptotic components. However, our IHC results showed a significant correlation of MLKL and p-MLKL expression in tumor tissues, and we also observed higher “spontaneous” cell death in MLKL-overexpression cell lines. We have used immunoblotting to analyze several insensitive cell lines treated with TSZ and found that p-RIP1 was upregulated while the p-RIP3 and p-MLKL could not be detected in these cell lines (data not shown), suggesting that a possible cause of necroptotic resistance in these cells could be the phosphorylation-abnormality of RIP3 or MLKL. These data suggest that the mechanisms regulating cellular sensitivity to necroptosis can be complicated and multifactorial. Further studies are needed to fully understand these mechanisms.

Apoptosis resistance has been widely recognized as one of the hallmarks of cancer cells. CASP8 has been reported by multiple researchers to be one of the most commonly mutated genes in HNSCC^13,43^. In consistency with these findings, we observed that TS treatment can only induce cell death in 4/11 cell lines. In addition, under the same concentration of TNFα and Smac mimetic, the proportion of cell death in TSZ group was significantly higher than that in TS group, which is similar to the findings on other cancer cell lines^34,35,44^, suggesting that some tumor cells with apoptotic resistance may be more sensitive to necroptosis. Several researchers have already adopted this concept and proved that targeting necroptosis is a plausible method that can bypass the apoptotic resistance and kill tumor cells^45–51^. Moreover, it was also reported to induce antitumor immunity in tumor microenvironment because of the release of pro-inflammatory DAMPs^21,22,52,53^. However, large amount of research is required to validate if this hypothesis can be adapted for HNSCC treatment.

We are the first study that reported necroptosis can be an independent prognostic marker for OS and PFS in HNSCC. In contradiction to our results, multiple previous research had demonstrated that MLKL is significantly downregulated in tumor tissues and low-level MLKL can be correlated with poor prognosis^17,54–58^. This may reflect the current paradoxical research status of necroptosis in cancers. With many researchers believed it as tumor suppressor that can be utilized for cancer treatment as above mentioned, others provided evidences that support it as potential cancer promoter. For example, Seifert et al.^37^ reported that necroptosis can induce the immunosuppressive microenvironment in PDA through CXCL1 and Mincle pathway which in turn promotes tumor invasion and metastasis. Seehawer et al.^59^ found that necroptotic microenvironment promoted oncogenically transformed hepatocytes develop into highly malignant intrahepatic cholangiocarcinoma, whereas the apoptotic microenvironment promoted its progression into mild hepatocellular carcinoma. In our study, we found that necroptotic cells could promote the migration and invasion of HNSCC cells by releasing DAMPs. And we further demonstrated IL- 1β as a necroptotic-specific DAMPs. Conos et al. have reported that necroptosis can induce the activation of NLRP3 inflammasome resulting in releasing of IL-1β^20^. Therefore, a possible explanation to our observation is that necroptotic IL-1β activates NF-κB pathway in treated tumor cells and further leads to increased migration and invasion. Although our results have clearly indicated that the necroptotic cells release DAMPs in a rather “active-secretion” manner, further research are needed to comprehensively address that how necroptosis affects the progression of HNSCC.

In conclusion, our study demonstrated that around half of the necrosis in HNSCC can be attributed to necroptosis and HNSCC cells have a higher tendency to develop necroptosis. Meanwhile, extensive necroptosis is an independent risk factor for poor OS and PFS in HNSCC. In addition, we constructed and verified a necroptosis cellular model in HNSCC. Finally, we demonstrated in our cellular model that necroptotic DAMPs can promote the migration and invasion of HNSCC cells. Our study provided novel insights in understanding necroptosis in HNSCC and might lay a foundation for future research. However, large amount of research are still needed to reveal the actual role of necroptosis in HNSCC.

## Supplementary information

Summary of supplementary files

Supplementary figure legends

Supplementary methods

Supplementary tables

Supplementary Figure 1

Supplementary Figure 2

Movie.S1_TS

Movie.S2_TSZ
