# Supplementary material for: Necroptosis in head and neck squamous cell carcinoma: characterization of clinicopathological relevance and in vitro cell model
Source: Cell Death Dis. 2020 May 22;11(5):391. doi: 10.1038/s41419-020-2538-5 (PMC7244585; doi:10.1038/s41419-020-2538-5)
Supplement: Supplementary file 1 — Summary of supplementary files [file 41419_2020_2538_MOESM1_ESM.docx]

**Text Summary**

**Supplementary methods.docx** contains supplementary methods & materials.

**Supplementary tables.docx** contains supplementary tables.

**Supplementary figure legends.docx** contains the figure legends for supplementary figures and movies.

**Movie. S1_TS.mp4** and **Movie. S2_TSZ.mp4** contain continuous imaging results of TS and TSZ treated cells.

**Supplementary Figure 1 300dpi.tif** contains the Supplementary Figure 1.

**Supplementary Figure 2 300dpi.tif** contains the Supplementary Figure 2.
