# Supplementary material for: Necroptosis in head and neck squamous cell carcinoma: characterization of clinicopathological relevance and in vitro cell model
Source: Cell Death Dis. 2020 May 22;11(5):391. doi: 10.1038/s41419-020-2538-5 (PMC7244585; doi:10.1038/s41419-020-2538-5)
Supplement: Supplementary file 2 — Supplementary figure legends [file 41419_2020_2538_MOESM2_ESM.docx]

**Supplementary figure legends**

**Movie. S1** Continuous imaging of SCC25 cells after TS treatment.

**Movie. S2** Continuous imaging of SCC25 cells after TSZ treatment.

**Fig. S1** (a) Representative images of different scores of p-MLKL. (b) Representative images of different scores of tumor necrosis. (c) Representative images of different scores of MLKL. (d) Kaplan-Meier survival analysis of the correlations between patients’ RFS and the level of MLKL or p-MLKL or tumor necrosis respectively. And the correlations between patients’ PFS and MLKL expression. The log-rank test was used to compare the survival rate between two groups, *p-value*<0.05 was considered significant.

**Fig. S2** (a) Quantification of AO-PI double staining in SCC25 and FaDu cells. Results are showed as the percentage of cells in each group. Fisher’s precise test was used to compare between three groups. ***, *P*<0.001. (b) Results of shRNA-knockdown assay in HT29 cells. The left panel shows the expression of p-RIP1, p-RIP3, p-MLKL and MLKL in shMLKL-HT29 and shNC-HT29 cells after 6 hours’ TSZ treatment. The right panel shows the mRNA expression of MLKL in shNC-HT29 and shMLKL-HT29 cells (Mean±SD, ***, *P*<0.001). (c) Transwell migration and invasion assays of SCC25 and FaDu cells treated with different conditioned medium for 24 hours. The bar graph shows the quantification results (Mean±SD, ***, *P*<0.001; **, *P*<0.01; *, *P*<0.05). (d) Transwell migration and invasion assays of HSC3 cells treated with different conditioned medium collected from SCC25 cells for 24 hours. The bar graph shows the quantification results (Mean±SD, ***, *P*<0.001; **, *P*<0.01; *, *P*<0.05). (e) Transwell migration and invasion assays of HSC6 cells treated with different conditioned medium collected from FaDu cells for 24 hours. The bar graph shows the quantification results (Mean±SD, ***, *P*<0.001; **, *P*<0.01; *, *P*<0.05). (f) Transwell migration and invasion assays of SCC25 cells treated with different conditioned medium collected from HT29 cells for 24 hours. The bar graph shows the quantification results (Mean±SD, ***, *P*<0.001; **, *P*<0.01; *, *P*<0.05).
