# Supplementary material for: Necroptosis in head and neck squamous cell carcinoma: characterization of clinicopathological relevance and in vitro cell model
Source: Cell Death Dis. 2020 May 22;11(5):391. doi: 10.1038/s41419-020-2538-5 (PMC7244585; doi:10.1038/s41419-020-2538-5)
Supplement: Supplementary file 3 — Supplementary methods [file 41419_2020_2538_MOESM3_ESM.docx]

**Supplementary methods**

**Continuous imaging of dying cells**

The SCC25 cells were seeded into 24 well plate at a concentration of 30 000 cells per well and left overnight to adhere. Cells were treated with TS and TSZ respectively, and the continuous imaging was performed using IncuCyte Live-Cell Analysis System following the instructions. After 19 hours scanning, the results were exported as videos.
