# Supplementary material for: Necroptosis in head and neck squamous cell carcinoma: characterization of clinicopathological relevance and in vitro cell model
Source: Cell Death Dis. 2020 May 22;11(5):391. doi: 10.1038/s41419-020-2538-5 (PMC7244585; doi:10.1038/s41419-020-2538-5)
Supplement: Supplementary file 4 — Supplementary tables [file 41419_2020_2538_MOESM4_ESM.docx]

**Table. S1** Correlation between MLKL and p-MLKL expression

| **p-MLKL expression** | **MLKL expression** | | | | **Total** | ***ρ***^*^ | ***P* value** |
| --- | --- | --- | --- | --- | --- | --- | --- |
|  | **None** | **Low** | **Medium** | **High** |  |  |  |
| **0** | 4 | 11 | 19 | 17 | 51 | 0.162 | **0.025** |
| **1** | 3 | 8 | 7 | 26 | 44 |  |  |
| **2** | 2 | 7 | 12 | 14 | 35 |  |  |
| **3** | 2 | 11 | 10 | 38 | 61 |  |  |
| **Total** | 11 | 37 | 48 | 95 | 191 |  |  |

*, Spearman rank correlation analysis, p<0.05 was considered statistically significant.

Changes：①The numbers in row “3” of old Table. S1 are moved to row “Total”. ②The actual numbers in row “3” are added. ③A “Total” column containing the sum of each row are added. Changes are marked in red in the table.
