# Supplementary figures and images for: Necroptosis in head and neck squamous cell carcinoma: characterization of clinicopathological relevance and in vitro cell model
Source: Cell Death Dis. 2020 May 22;11(5):391. doi: 10.1038/s41419-020-2538-5 (PMC7244585; doi:10.1038/s41419-020-2538-5)

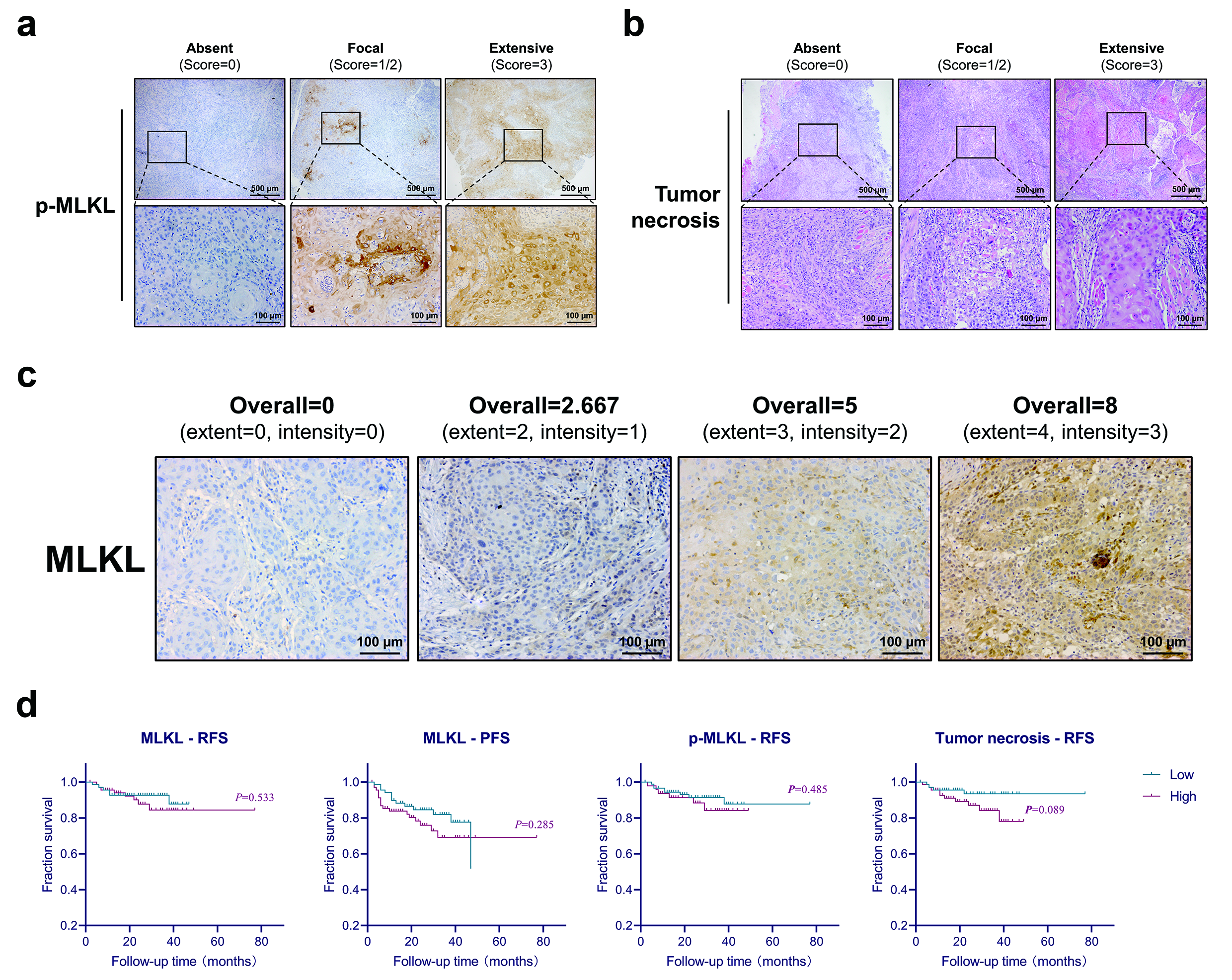

Supplement: Supplementary file 5 — Supplementary Figure 1 [file 41419_2020_2538_MOESM5_ESM.tif]

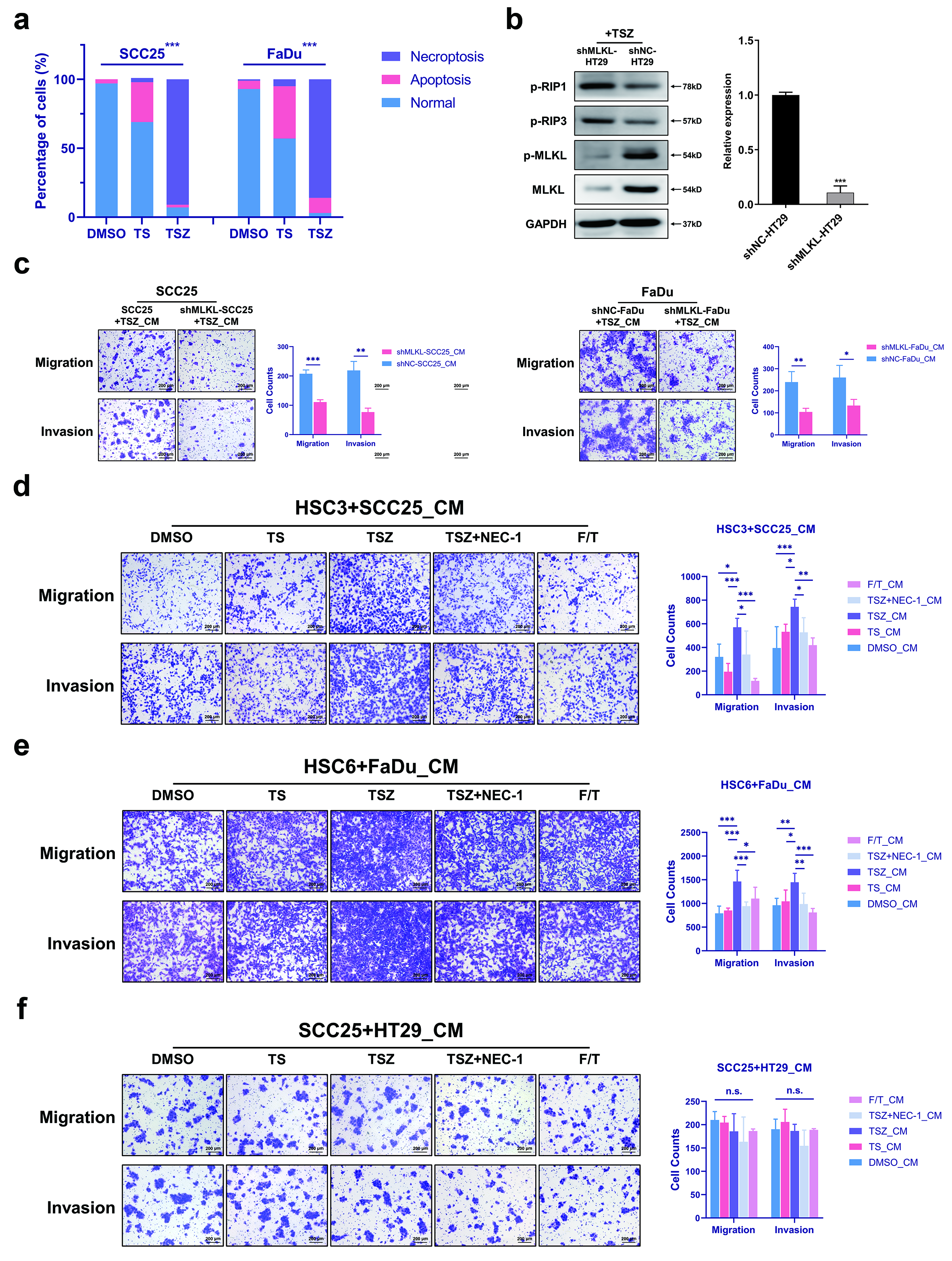

Supplement: Supplementary file 6 — Supplementary Figure 2 [file 41419_2020_2538_MOESM6_ESM.tif]
